# Mineralosphere Microbiome Leading to Changed Geochemical Properties of Sedimentary Rocks from Aiqigou Mud Volcano, Northwest China

**DOI:** 10.3390/microorganisms9030560

**Published:** 2021-03-09

**Authors:** Ke Ma, Anzhou Ma, Guodong Zheng, Ge Ren, Fei Xie, Hanchang Zhou, Jun Yin, Yu Liang, Xuliang Zhuang, Guoqiang Zhuang

**Affiliations:** 1 Research Center for Eco-Environmental Sciences, Chinese Academy of Sciences, Beijing 100085, China; make.1997@163.com (K.M.); feixie_st@rcees.ac.cn (F.X.); hczhou_st@rcees.ac.cn (H.Z.); yin_yinjun@foxmail.com (J.Y.); liangyu_0216@163.com (Y.L.); xlzhuang@rcees.ac.cn (X.Z.); 2 College of Resources and Environment, University of Chinese Academy of Sciences, Beijing 100049, China; 3 Sino-Danish College, University of Chinese Academy of Sciences, Beijing 101400, China; 4 Sino-Danish Center for Education and Research, Beijing 101400, China; 5 Key Laboratory of Petroleum Resources, Institute of Geology and Geophysics, Chinese Academy of Sciences, Lanzhou 730000, China; gdzhbj@mail.iggcas.ac.cn; 6 National Institute of Metrology, Beijing 100029, China; reng@nim.ac.cn

**Keywords:** microbial community, iron-bearing mineral, hydrocarbon, organic-inorganic interaction, mud volcano

## Abstract

The properties of rocks can be greatly affected by seepage hydrocarbons in petroleum-related mud volcanoes. Among them, the color of sedimentary rocks can reflect the changes of sedimentary environment and weathering history. However, little is known about the microbial communities and their biogeochemical significance in these environments. In this study, contrasting rock samples were collected from the Aiqigou mud volcano on the southern margin of the Junggar Basin in Northwest China as guided by rock colors indicative of redox conditions. The physicochemical properties and mineral composition are similar under the same redox conditions. For example, the content of chlorite, muscovite, quartz, and total carbon were higher, and the total iron was lower under reduced conditions compared with oxidized environments. High-throughput sequencing of 16S rRNA gene amplicons revealed that different functional microorganisms may exist under different redox conditions; microbes in oxidized conditions have higher diversity. Statistical analysis and incubation experiments indicated that the microbial community structure is closely related to the content of iron which may be an important factor for color stratification of continental sedimentary rocks in the Aiqigou mud volcano. The interactions between organics and iron-bearing minerals mediated by microorganisms have also been hypothesized.

## 1. Introduction

Due to the existence of natural gas seepage in the mud volcano systems, the reduction of minerals and/or elements likely became the dominant process, which has received a great deal of attention [1]. Natural gas seepage is the steady or intermittent, slow or rapid, visible or invisible flow of hydrocarbon gases such as methane, ethane, and propane from subsurface sources into the atmosphere [2]. Mud volcanoes are the most extensive surface expressions of hydrocarbons fluid migration in petroleum bearing sedimentary basins [3]. Although studies have shown that naturally geological methane emissions are an order of magnitude (1.6–5.4 tera grams per year) lower than currently used estimates (10–20 tera grams per year) [4], mud volcanoes are still an important source for the contribution, approximately 30%, of the geological methane emissions [5].

Long-term leakage of hydrocarbons can produce near-surface oxidation-reduction zones, which are conducive to the development of a variety of chemical and mineralogical changes [6]. Such changes can be observed in the surface color, structure, and hardness of the affected rocks [7]. Among them, the color change of sedimentary rocks is widely regarded as a good indicator of their depositional environments, diagenesis, and weathering history [8]. Surface seepage of geological fluids, such as crude oils and natural gases from mud volcanoes can transform the color of rocks from bright reddish to green, deep gray, or black, and is known as the bleaching effect [7,9]. The bleaching of rocks surrounding mud volcanoes indicates multiple organic–inorganic interactions occurring within fluids, leading to complex chemical reactions between the geological fluid itself and the surrounding minerals [1]. The most important factor determining the color of rocks is iron and its speciation [10]. The dominant presence of ferrous iron (generally visible as gray and green colors) is indicative of a reduced environment, conducive for the formation of potential petroleum source rocks, whereas iron-bearing minerals rich in ferric iron (visible as red or yellow colors) are indicative of oxidized conditions [8].

According to previous studies, only an abiotic process has been mentioned [7,9,11]. However, the metabolic activity of microorganisms can also affect the valence of iron, and microorganisms likely play a role in the color stratification of continental sedimentary rocks. Microorganisms release and store energy through redox reactions by oxidizing organic matter, combined with electron acceptor reduction of other compounds, such as humus, iron-bearing minerals, and transitional metals [12]. Microbial colonization and growth can lead to a range of effects in the environment, including mineral discoloration and staining, deterioration, and even mineral transformations through the redox transformations of various elements, e.g., Fe, S, and Mn [13]. A number of microorganisms can produce nonspecific and specific iron chelators as iron binding and iron transporter compounds, thus affecting the formation, dissolution, and transition of mineral phases. In general, microbes in mud volcanos may be involved in the process of iron/sulfur-dependent color changes because of the potential hydrocarbon reservoir as well as the high iron concentration. This redox process mediated by geological microorganisms not only plays a vital role in the formation and dissolution of mineral phases but also closely relates to the generation, preservation, destruction, development, and exploration of oil and gas resources [14]. A large number of anaerobic methanotrophs and various sulfate- and iron-reducing microorganisms participate in the redox reaction of methane coupled with iron and sulfur in mud volcanos, thus promoting the biogeochemical cycling [15].

Differently colored rock formations may contain distinct microbial groups carrying out various metabolic processes of potential biogeochemical significance. Hence, minerals in rocks and other places are conducive for the development of specific microbial communities based on their mineralogy, nutrient content, and weatherability. This microbial habitat is defined as the “mineralosphere” [16]. However, little scientific attention has been given to the influence of redox conditions on microbiome composition in solid rocks.

In this study, six contrasting rock samples with different redox conditions were collected from the Aiqigou mud volcano on the southern margin of the Junggar Basin in Northwest China. The main objectives of this study were (1) to explore the microbial community structure and physicochemical properties under different redox conditions and (2) to investigate whether microbes are involved in color stratification in this area and their potential role in redox reactions.

## 2. Materials and Methods

### 2.1. Site Description and Sample Collection

**Figure 1:**
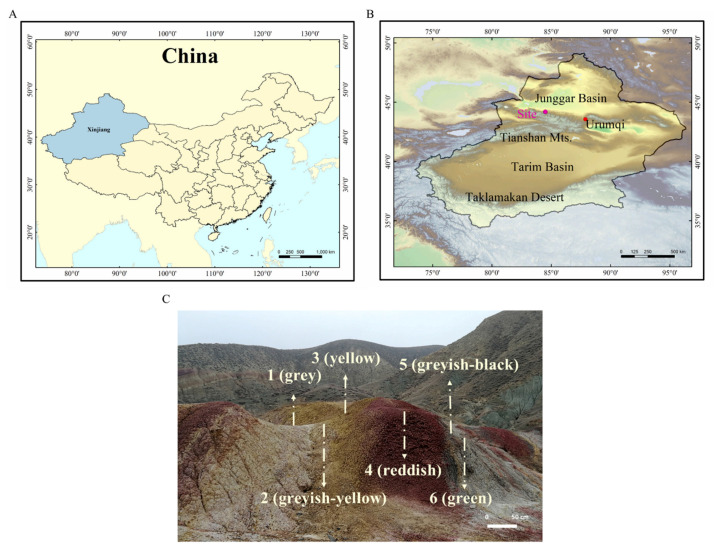
Location of research sites along the southern margin of the Junggar Basin. (**A**) The blue shading shows the boundary of Xinjiang Uyghur Autonomous Region. (**B**) Some of the characteristic sites and our sampling site are shown in the Xinjiang Uyghur Autonomous Region. (**C**) The geological features of the six colored layers sampled. AQG1: gray, AQG2: grayish-yellow, AQG3: yellow, AQG4: reddish, AQG5: grayish-black, AQG6: green.

The Junggar Basin, one of the largest Mesozoic–Cenozoic sedimentary basins in Northwest China, is a major petroliferous basin in Xinjiang (Figure 1A), covering an area of 1.3 × 10^5^ km^2^ [17]. In the south margin of this basin, numerous mud volcanoes (groups) such as Anjihai, Dushanzi, Aiqigou, and Baiyanggou are successively distributed from east to west [15]. The strata exposed by the Aiqigou mud volcano are Cretaceous rocks [18]; natural gas and oil in underground reservoirs diffuse to the surface and then react with the exposed rocks which contain iron-bearing minerals. Therefore, the Aiqigou area provides a suitable environment to study microbial-mediated fluid–mineral interactions.

Six terrestrial rocks (Figure 1C) of varying colors (AQG1: gray, AQG2: grayish-yellow, AQG3: yellow, AQG4: reddish, AQG5: grayish-black, AQG6: green) in Aiqigou were collected on 11 November 2018, on the northern piedmont of the Tianshan Mountains (Figure 1B) (44° 11′27.04″ N, 084°29′54.23″ E). The collected samples showed visible color differences on the surface, with bright reddish on top of the hills and gradually becoming lighter at the bottom (Figure 1C). Rock samples AQG2/3/4 were considered to be under oxidized environments, whereas samples AQG1/5/6 were considered to represent reduced environmental conditions. The difference in the color of these rocks has previously been shown to be caused by the bleaching effect of crude oil and natural gas, as well as the activity of previous mud volcanoes [1,9]. After a rough scraping of the rock surface, solid cores were taken from 5 to 20 cm in depth using a shovel. Samples from each site, weighing more than 2 kg each, were sealed in a box containing dry ice for transport to the Research Center for Eco-Environmental Sciences within 48 h. The shovel and box were sterilized by 75% alcohol, only one sample was taken in one layer. Samples were stored in a −20 °C freezer for 16S rRNA gene amplicon high-throughput sequencing and mineralogical analysis.

### 2.2. Rock Physicochemical Analyses

All sediment samples were crushed in an agate mortar and pestle, then passed through a 100-mesh sieve, mixed evenly, and finally stored in dry and hermetic conditions to avoid contamination and minimize chemical changes. pH was measured in a 1:5 solid-to-deionized water mixture by using an electrode (Mettler Toledo, Switzerland). Total carbon (TC) and total nitrogen (TN) of the sample were measured by an elemental analyzer (Vario MACRO cube, Elementar, Germany). The concentrations of ammonium nitrogen (NH_4_^+^) and nitrate nitrogen (NO_3_^−^) were measured in a 1:5 solid-KCl (2 mol L^−1^) mixture filtered through a 0.45 μm membrane (Merck KGaA) by the AA3 flow analyzer (SEAL-AA3, Germany). Inductively coupled plasma optical emission spectrometry (ICP-OES, Shimadzu 9820, Kyoto, Japan) was used to detect total iron and manganese concentration, the medium was HNO_3_ (1%). The data were statistically analyzed by one-way analysis of variance (ANOVA) and multiple comparative tests for mean separation were performed using the post-hoc Tukey’s HSD test [19]. These data were obtained by using the IBM SPSS 25 software. T-test analysis was carried out by Excel.

The mineral composition of the samples was determined by powder X-ray diffraction without any chemical pretreatment. Conventional XRD was performed on a D/max-RB diffractometer (Cu Kα radiation; Rigaku, Rotafles, Japan). The sample was scanned at 2 deg/min for every 0.02°(2θ) step in the 3–70°(2θ) interval. The divergence, scattering, and receiving slits were 0.5°, 0.5°, and 0.15 mm, respectively.

The ARL Perform X 4200 roentgen X-ray fluorescence spectrometer (Thermo Fisher, Greenville, SC, USA) was used to analyze the major element compositions. The spectrometer was operated at 50 kV and 50 mA. The samples were prepared by mixing about 2 g powder with boric acid and compressed in a tablet press for subsequent XRF measurement. Loss on ignition (LOI) was determined by heating the powder samples in an oven at 80 °C for 4 h and then in a Muffle furnace at 850 °C for 6 h.

### 2.3. Enrichments

To test the activity of iron reduction and methane oxidation in the mud volcano, microcosms were established with 5 g of powdered samples and 5 mL sterile water in 35 mL sterile serum bottles for pre-incubation. Bottles were sealed with butyl rubber stoppers, evacuated, and flush with Ar 4 times, 5 min each time [15]. Finally, the headspace was filled with Ar and kept at 1 bar atmospheric pressure at 30 °C for 7 days to activate microorganisms in the sample and deplete residual acceptors [20].

Amorphous iron was prepared as previously described [21], and 2.5 mL amorphous iron (400 mM) and 2.5 mL sodium acetate (400 mM) were added into individual serum bottles under anaerobic conditions after pre-incubation to promote the growth of iron-reducing bacteria, then sealed, evacuated, and flushed with Ar as before. After the last evacuation, 26 mL of an Ar: ^13^CH_4_ (80:20) gas mixture was injected into the headspace for incubation. Triplicate enrichments were used per rock layer, and one autoclaved control experiment was set up for each layer to verify the microbial-mediated processes of iron and hydrocarbons. All the microcosms were incubated at 30 °C, destructive sampling was conducted every 7 days to measure Fe^2+^, CH_4_, and CO_2_ concentrations.

Ferrous iron was determined by the Ferrozine method [22]. A gas chromatograph coupled with a mass spectrometer (GCMS-QP2010 Ultra, Shimadzu, Kyoto, Japan) was used to measure the concentration of CH_4_ and CO_2_. Stable isotope compositions of the carbon (δ^13^C values) were tested using a Delta V Advantage gas chromatography combustion isotope ratio mass spectrometer (GC-C-IRMS, Thermo Fisher Scientific, Bremen, Germany) [15].

### 2.4. DNA Extraction, Amplification, and High Throughput Sequencing of the 16S rRNA Gene

DNA was extracted from the powered samples of each layer in triplicate to reduce sampling error. PowerSoil DNA Isolation kits (MoBio Laboratories, Inc., Carlsbad, CA, USA) were used for DNA extraction. After extraction, the purity and concentration of DNA were determined by NanoDrop 2000 (Thermo Scientific, Wilmington, DE, USA), and the extracted DNA was placed in a −20 °C refrigerator for subsequent amplification and sequencing.

For high-throughput sequencing, the V4-V5 highly variable region of the 16S rRNA gene was selected for amplification, with primers 515F (5′-GTGCCAGCMGCCGCGGTAA-3′) and 907R (5′-CCGTCAATTCCTTTGAGTTT-3′) [23]. The reaction system was 50 μL, including 2 × Taq PCR mix (25 μL), 1 μL forward primer (10 μM), 1 μL reverse primer (10 μM), 3 μL DNA, and sterile deionized water (20 μL). The PCR amplification procedure was initial denaturation at 95 °C for 5 min, 34 cycles of denaturation at 94 °C for 1 min, primer annealing at 57 °C for 45 s, then extension at 72 °C for 1 min. Finally, it was extended at 72 °C for 10 min. PCR products were detected by electrophoresis on a 2% agarose gel. Ion Plus Fragment Library Kit 48 RXNS from Thermofisher company was used for library construction, and sequencing was performed on the Ion S5^TM^XL platform. The commitment error rate of Ion S5^TM^XL sequencing platform is Q20 (1%). All the sequence data have been submitted to GenBank sequence read files (http://www.ncbi.nlm.nih.gov, accessed on 1 September 2020), the BioProject ID is PRJNA638372.

### 2.5. Quantitative PCR

The CFX Connect Real-Time System (BioRad, Hercules, CA, USA) instrument was used to conduct quantitative PCR for the 16S rRNA gene of archaea and bacteria in the sedimentary rocks from the different rock layers. Quantitative PCR analyses were performed with a Takara SYBR^®^ Premix Ex TaqTM kit (Takara, Dalian, China).

The reaction system was 25 μL, including 12.5 μL TB Green Premix ExTaq II Mix (2×), 0.5 μL forward primer (10 μM), 0.5 μL reverse primer (10 μM), 2 μL DNA (5 ng μL^−1^), and 9.5 μL sterile deionized water. The primers used for the quantitative determination of archaea were A806F (5′-ATTAGATACCCSBGTAGTCC-3′) [24] and A958R (5′-YCCGGCGTTG AMTCCAATT-3′) [25]. The primers used for bacteria were BAC 27F (5′-AGAGTTTGATCCTGGCTCAG-3′) [26] and EUB 338R (5′-GCTGCCTCCCGTAGGAGT-3′) [27]. The reaction procedure was as follows: (1) 95 °C for 30 s; (2) 40 PCR cycles of 95 °C for 5 s, annealing (bacteria: 56 °C for 45 s, archaea: 60 °C for 30 s), then 72 °C for 45 s, forming the melt curve. Standards were prepared as a 10-fold dilutions of a known copy number of plasmid DNA. Three replicates were performed for each group. The quantitative real-time PCR (qPCR) efficiency of the standard curve was 103.5% in archaea and 104.4% in bacteria, and R^2^ was 0.998 in archaea and 0.995 in bacteria. Quantification results in units that are converted to copies numbers per gram of powdery sediment.

The preparation of standard plasmid includes amplification of specific sequence, connection and transformation of plasmid, extraction of plasmid DNA, and conversion of copy number. The specific steps are as follows:

(1) The primers were the same as above, and the sequences were synthesized by Sangon Biotech. Takara Taq^TM^ Hot Start Version (Takara, Dalian, China) was selected as the system. The reaction system was 25 μL: 1 μL forward primer (10 μM), 1 μL reverse primer (10 μM), 1 μL DNA (10–40 ng μL^−1^), 0.2 μL Takara Taq HS, 2.5 μL 10× PCR Buffer, 1.5 μL dNTP mixture and the remaining volume was replenished with sterile deionized water. The procedure recommended in the instructions was used for the reaction, and the PCR products obtained after amplification were verified by 1.5% agarose gel electrophoresis and then recycled by gel cutting by AxyPrep DNA Gel Extraction Kit (Axygen Biosciences, Tewksbury, MA, USA).
(2) The purified PCR products were sequenced and cloned into the pGEM-T Easy Vector (Promega, Madison, WI, USA), and then transformed into the *Escherichia coli* DH5α (TIANGEN, Beijing, China) [19]. The obtained strains were verified by PCR and sequencing, and the DNA length was calculated by BioEdit.
(3) The plasmids with target genes were extracted with TIANprep Mini Plasmid Kit (TIANGEN Biotech, Beijing, China), then the Plasmid DNA was used NanoDrop 2000 (Thermo scientific, Wilmington, DE, USA) to determine the purity and concentration. A260/280 between 1.8–2.0 indicates that the plasmid is of good quality and can be used in the production of standard curves. According to the concentration, the copy number of the plasmid can be calculated according to Equation (1):

(1) $$\mathrm{copy}\;\mathrm{number}\;\left({\mathrm{copies}\;g}^{-1}\;\mathrm{sediment}\right)=\frac{6.02\times 10^{23}\times C}{\mathrm{DNA}\;\mathrm{length}\times 660}$$

where 6.02 × 10^23^ is Avogadro’s constant, *C* is the concentration of DNA, the length of DNA is 3326 bp (Bacteria) or 3167 bp (Archaea), and 660 is the average molecular weight of each nucleobase of double-stranded DNA.

### 2.6. Data and Statistical Analyses

DNA sequences were processed using a Galaxy pipeline (http://mem.rcees.ac.cn:8080/ (accessed on 7 August 2019)) [28,29] integrated with various bioinformatics tools. The raw sequences were assigned to their respective samples using the barcodes and trimmed, and then paired end reads were merged into full length through FLASH [30], and the unqualified sequences were filtered out using the program Btrim [31]. UPARSE was used to remove chimers and to classify operational taxonomic units (OTUs) at 97% similarity level, followed by random re-sampling. The RDP classifier method [32] was employed to assign prokaryotic sequences to different taxa in the SILVA database 132 version.

Chao and Shannon indices were used to assess the differences in species richness and diversity of microbial communities in each sample. The heterogeneity of microbial community composition at the six layers was evaluated using non-metric multidimensional scaling (NMDS). Mantel tests were used to estimate the relationship between bacterial community structure and each environmental variable [20]. Canonical correlation analysis (CCA) was used to reflect the impact of environmental factors on different communities [32].

Path analysis was used to evaluate the relationships between microbial communities, microbial diversity, species richness, and iron and other environmental factors. The Shannon index was taken into the model as an indicator of microbial diversity and the Chao value was used to represent species richness. Principal component analysis (PCA) was performed on the resampled OTU table on this web page (http://mem.rcees.ac.cn:8080/ (accessed on 7 August 2019)), and the first principal coordinate axis (PC1) obtained was taken into the model as an indicator of microbial community [20]. Other environmental factors were selected and extracted as the first principal component (PC1), which was driven by TC, TN, NO_3_−, NH4+, Mn, and pH. The final model was generated to match data and model fitting index (the model had a good fit when χ^2^ < 2 and *p* > 0.05). All path analyses were conducted using an online program called SPSSAU application (https://www.spssau.com (accessed on 1 March 2020)).

## 3. Results

### 3.1. Mineralogical Compositions

**Figure 2:**
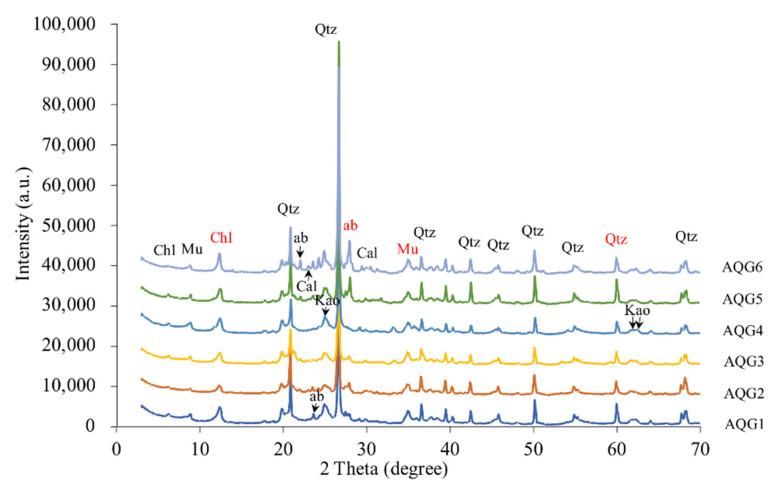
X-ray diffraction patterns for the six sampled rock layers. Chl—chlorite; ab—albite; Mu—muscovite; Qtz—quartz; Cal—calcite; Kao—kaolinite. The red font is mainly described in the text.

XRD analyses revealed identifiable variations in mineral components and relative content for the sampled rock layers (Figure 2). A wide, strong peak representing smectite in the original rocks was not present in any of the bleaching layers. In contrast, chlorite (2 Theta = 12.38) and muscovite (2 Theta = 34.98) were found in all layers, but their peaks under reduced conditions (AQG1 and AQG6) were higher than those under oxidized conditions (AQG2 and AQG3). Kaolinite was present in all layers, but the peak shift and the magnitude of the peak differed among rock layers, probably indicating some changes in the content and crystal structures were altered during the bleaching process. For felsic minerals, the peaks of quartz (2 Theta = 60.14) were weaker under AQG2, AQG3, and AQG4 layers, however, another felsic mineral, albite (2 Theta = 27.95), appeared only in the AQG5 and AQG6. These mineralogical compositions were likely modified due to dissolution and removal as geofluids passed through the rock.

### 3.2. Chemical Composition of Major Elements

The mineralogical compositions of the rock materials were analyzed by XRF analysis and are shown in Table 1. SiO_2_ was prominently enriched in all layers, accounting for 55.68–62.39 wt.%, followed by Al_2_O_3_, accounting for 20.28–27.29 wt.%, and Na_2_O varied from 1.03–6.22 wt.%. Higher sodium content probably reflects the increase of albite and related clay minerals in AQG5 and AQG6. Fe_2_O_3_ was 6.15–9.76% in oxidized rocks (AQG2/3/4) as opposed to only 3.90–5.26% in the reduced layers (AQG1/5/6). Elements such as magnesium, manganese, and calcium are considered as carbonate-forming elements and were relatively rare. These results are also reliably supported by the XRD analysis (Figure 2). P_2_O_5_ was abundant in the relatively oxidized rocks that appeared reddish or yellow (AQG3: 0.143 wt.% and AQG4: 0.160 wt.%). In addition, loss on ignition (LOI) values were clearly higher in AQG1 (0.538 wt.%) and AQG5 (0.615 wt.%).

### 3.3. Microbial Communities in Different Rock Layers

**Figure 3:**
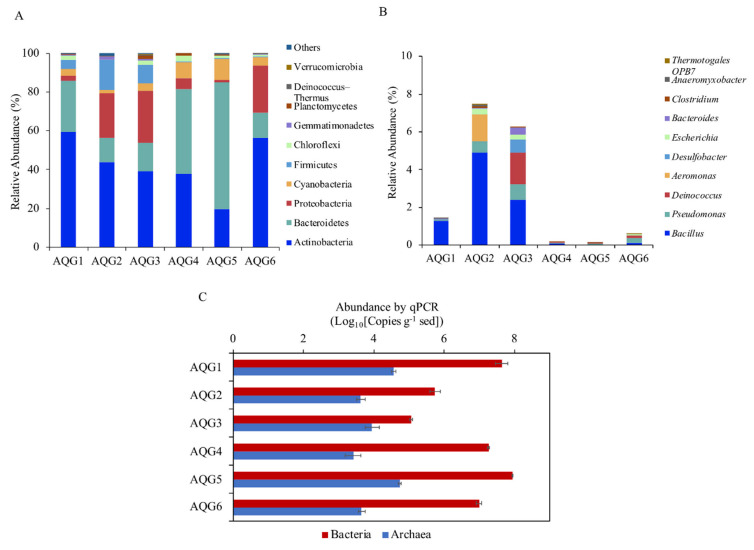
(**A**) Relative abundance of prokaryotes at phylum level (top 10) in the six rock layers. “Others” is the sum of the relative abundances at phylum level out of the top 10. (**B**) Relative abundance of iron-reducing bacteria at the genus level (top 10) in the six rock layers. Mean relative abundance of microorganisms in three parallel samples taken from each rock. The height of each column represents relative abundance, and the color represents a particular phylum or genus. (**C**) 16S rRNA gene copy numbers of archaea and bacteria in the six rock layers. (**A**–**C**) The value indicates the means of 3 replicates and error bars are represent standard deviations.).

A total of 1,525,076 high-quality sequences were obtained. After random resampling, 76,312 sequences per sample were used to compare the prokaryotic communities in different rock layers. A total of 1421 operational taxonomic units (OTUs; ≥97% sequence similarity) were obtained. The bacterial and archaeal 16S rRNA gene copy numbers in the six rock layers were analyzed by qPCR and ranged from 1.16 × 10^5^ to 8.71 × 10^7^ and 2.59 × 10^3^ to 5.45 × 10^4^ copies per g of sediment (Figure 3C). Archaea accounted for 0.01% to 7.18% of the total prokaryotes. The rarefaction curves were nearly saturated, indicating that the sequencing results provided good coverage (Figure S1). The figure of melting curves of standards and samples was provided to demonstrate that SYBR was a valid alternative to TaqMan probe technology (Figure S2). Standard curves of standards and samples are shown in Figure S3.

A total of 2 archaeal phyla and 33 bacterial phyla were identified, with 18 phyla being present in all rock layers. Figure 3A depicts the top 10 phyla with the highest relative abundance in each rock and shows that contrasting communities inhabited the different rock layers. The phylum with the highest relative abundance across the entire dataset was Actinobacteria, ranging from 19.5% to 59.3%, followed by Bacteroidetes and Proteobacteria, which ranged from 12.7% to 65.3% and 1.43% to 26.84%, respectively. Other phyla such as Cyanobacteria, Firmicutes, Chloroflexi, and Gemmatimonadetes were also present in all rock layers, but in lower abundance. The archaeal sequences belonged to the phyla Thaumarchaeota with the highest in AQG3 (0.18%) and Euryarchaeota with the highest relative abundance in AQG5 (0.30%).

The relative abundance of the top 10 genera of iron-reducing bacteria is shown in Figure 3B. These 10 genera accounted for about 97% of the known iron-reducing bacteria in the samples. The remaining genus-level classifications displayed a great deal of variance from one rock layer to another. Among them, the relative abundance of *Bacillus* (Firmicutes) was the highest at all of the sites (0.03–4.90%). The other genus with high abundance were *Pseudomonas*, accounting for 0.05–0.80%. In addition, the relative abundance of iron-reducing bacteria in AQG2 was the highest, followed by AQG3.

**Figure 4:**
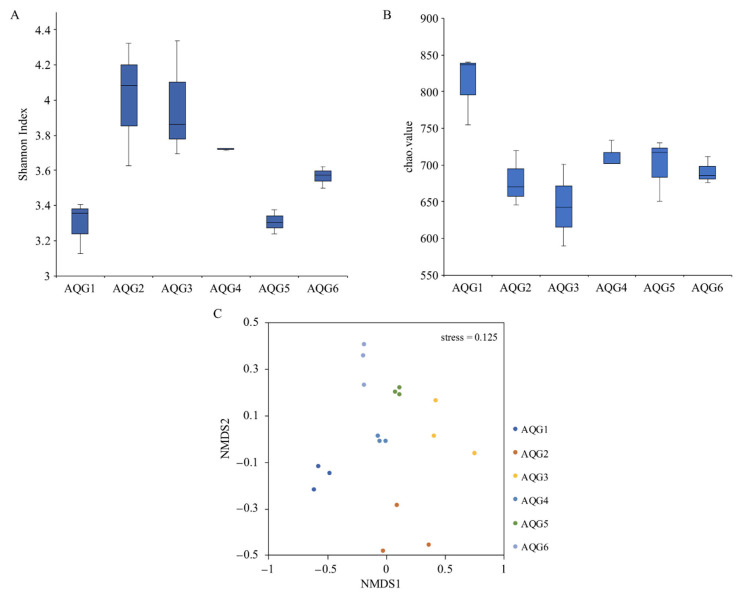
(**A**,**B**) Alpha diversity of microbial communities in the six rock layers in the Aiqigou (AQG) mud volcano. Each value indicates 3 replicates values and error bars represent standard deviations. (**C**) Non-metric multidimensional scaling analysis describing the microbial community structure of each layer is presented along two major dimension-reduced axes based on Bray-Curtis distance. (Stress < 0.2, non-metric multidimensional scaling (NMDS) can accurately reflect the degree of difference between samples).

Weighted UniFrac distance matrix was used for UPGMA clustering analysis, as shown in Figure S4. Rock samples AQG2, AQG3, and AQG6 were clustered together, AQG1 and AQG4 were clustered together. AQG5 was obviously different from other layers. We also assessed the microbial community structure of the six different sample layers by community diversity analysis. Shannon indices are commonly used to reflect microbial diversity (Figure 4A). This meant that oxidized conditions such as AQG2 (4.01), AQG3 (3.97), and AQG4 (3.72) have higher diversity (mean value). The greater the Chao1 value, the higher the species richness (Figure 4B). The results of alpha diversity analysis indicated that the richness was highest in AQG1 (810.7), followed by AQG4 (712.4) and AQG5 (699.1), whereas the lowest level was found in AQG3 (644.0) (mean value). Significance results by *t*-test between each group were shown in Table S1. Non-metric multidimensional scaling (NMDS) analysis showed microbial communities between different sites (Figure 4C), with the six sample layers clustering individually. AQG2/4/5/6 was similar in the first scaled dimension, while AQG1 and AQG3 were separated from other samples along this first scale. Along the second scaled dimension, AQG2 was separated from the other samples.

### 3.4. Environmental Factors Associated with Microbial Community Structure

The environmental properties are shown in Table S2. All rock layers were weakly alkaline, the pH range was 7.51–8.83. The concentration of ammonium and nitrate was different in each layer. Site AQG6 presented extremely low ammonium concentration and followed by AQG3 and AQG2, with values of 0.97, 2.77, and 7.90 mg kg^−1^, respectively. The nitrate concentrations ranged from 22.05 to 231.94 mg kg^−1^, with the highest in AQG5 and the second highest in AQG3. The content of total nitrogen was similar among the samples. Total carbon (TC) content ranged from 1.13 to 8.87 g kg^−1^, among which the TC content in the reduced rock samples (AQG1/5/6) was higher compared with the other layers (AQG2/3/4). Among the two measured redox-sensitive metals (Fe and Mn), Fe was abundant in the relatively oxidized rocks (AQG2/3/4), among 34.22–50.66 g kg^−1^, Mn was relatively less abundant in all layers. The multiple comparisons result of the HSD test of physicochemical properties of different layers is shown in Table S3, most of them were significantly correlated with each other (*p* < 0.05).

The Mantel tests identified environmental factors that drove community changes (Table S4). All measured environmental factors were significantly correlated with the β-diversity of the prokaryotic communities (Bray-Curtis distance; simple Mantel tests, *p* < 0.05). In order to further clarify the influence of the individual environmental variables versus the other environmental factors, partial Mantel tests were performed (Table S4), NO_3_^−^ and iron were the key environmental factors that had a significant impact on the microbial community (*p* < 0.001). These results were well supported by the individual ANOVA test (Table S5) and CCA result (Figure S5), where all rock layers are clustered separately. The relationship between microbial community and environmental factors were clarified. The transformed data samples were plotted based on the two coordinates CCA1 and CCA2 according to the environmental variables (Figure S5). The results showed that Fe had a higher effect on the samples obtained from the right platform (AQG6), followed by a very slight effect on the TN value, while Mn and nitrate had a positive effect on the AQG3 and AQG2. pH was a minor determinant of the microbial community structure of the samples obtained from top regions (AQG1, AQG4, and AQG5). In general, Fe and nitrate were the major influencing factors.

### 3.5. Biogeochemical Process of Hydrocarbon and Iron in Enrichments

**Figure 5:**
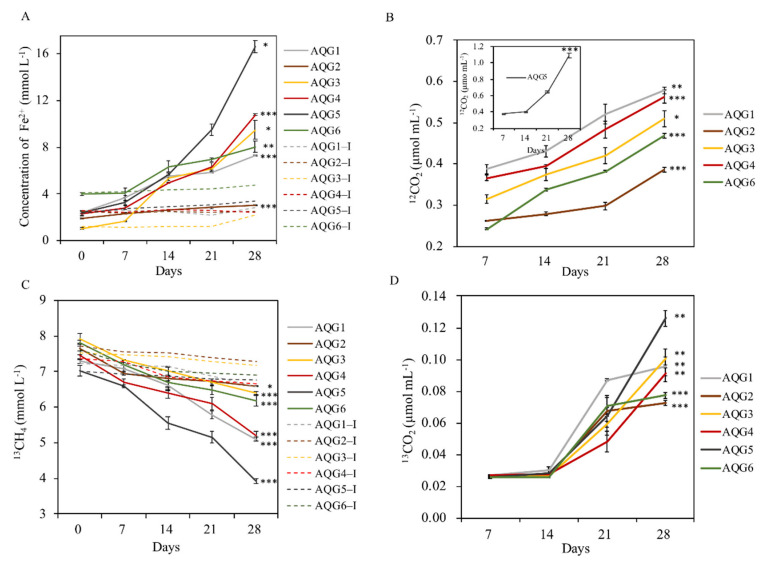
(**A**) Ferrozine-extractable Fe (II) in enrichments. (**B**,**D**) The production of ^12^CO_2_ and ^13^CO_2_. (**C**) Anaerobic oxidation of ^13^CH_4_. The dotted line represents the heat-inactivated control group. Error bars represent one standard deviation of the mean (*n* = 3). The asterisk represents the significance of the *t*-test results between the 28 day and the initial data (0 day in Figure 5A,C, 7 day in Figure 5B,D) in experimental group (*** *p* < 0.001, ** *p* < 0.01, * *p* < 0.05).

There were some differences in metabolic processes such as iron reduction potential and methane anaerobic oxidation rate among the different rock layers (Figure 5). The results showed that the content of gas and iron in all experimental groups had significant changes after enrichment culture (*p* < 0.05). Among them, AQG5 had the highest iron reduction rate, with ferrous iron accumulation reaching 16.6 mmol L^−1^ at 28 days of incubation. AQG2 had the lowest iron reduction rate, with a ferrous iron accumulation of only 3.0 mmol L^−1^. In addition, the trend of the inactivated control group was basically stable or slightly increased, indicating that microorganisms did participate in the reaction process of iron reduction. The yield of ^12^CO_2_ was used to characterize the oxidation rate of organic matter in various layers. The yield of ^12^CO_2_ in AQG5 was 1.1 μmol mL^−1^, which was much higher than that in the other 5 layers. In addition, the consumption of ^13^CH_4_ in AQG5 was the fastest, followed by AQG1 and AQG4, while the content of ^13^CH_4_ in the control group was only slightly reduced. The production of ^13^CO_2_ was used to characterize the anaerobic oxidation rates of methane in various layers, which ranged from 0.026 to 0.126 μmol mL^−1^ in AQG5. The contents of ^12^CO_2_ and ^13^CO_2_ in the control inactivated group were lower than the minimum detection line, so they were ignored.

## 4. Discussion

### 4.1. Geochemical Properties of AQG Terrestrial Rocks

As with previous results, quartz, albite, chlorite, muscovite, and kaolinite were ubiquitous composition in this mineral assemblage [7,9]. As illustrated in Figure 2, smectite was not present in all layers but was found in other unbleached areas far from the oil and gas seepage [1,7]. The peaks of chlorite and muscovite under reduced conditions (AQG1/5/6) were higher than those under oxidized conditions (AQG2/3/4). These differences may indicate that during the process of bleaching, smectite has been converted into other minerals such as chlorite and muscovite [1,7]. Additionally, microbial activity may also play a vital role. Previous studies showed that under various conditions, expansive smectite was readily reduced by bacteria [33]. Microorganisms could promote the transition from smectite to illite, an intermediate mineral between smectite and muscovite, within two weeks at room temperature and one atmosphere [34]. Therefore, under the strong reducing conditions of the bleached layers (AQG1/5/6), bacteria may enhance mineral transformation, thus increasing the abundance of chlorite and muscovite.

As a secondary mineral, kaolinite is the weathering product of feldspar or other silicate minerals in the process of soil formation. The peak intensity of kaolinite was higher under reduced conditions (AQG1/5/6) than under oxidized conditions (AQG2/3/4), which was different from the normal phenomenon and may indicate the importance of microbial processes. Some of the microorganisms that inhabit rocks could also be associated with rock weathering by promoting mineral dissolution and diagenesis [35], leading to this unusual result. In addition, certain bacteria can cause mineral weathering alone by interacting with various minerals [36] and the weathering may provide a variety of nutrients for life in lithologic environments [37]. Together these results implied that microorganisms could enhance the mineral weathering and increase the content of kaolinite under reduced conditions (AQG1/5/6).

For felsic minerals, clay mineral conversion may be one of the sources of silica [38]. It was found that during the transformation from smectite to illite and from illite to muscovite in the diagenetic environment, a large amount of silicon was released [39], which could improve the formation of quartz [40,41]. Siliceous organisms [38] and feldspar alteration [42] may also affect the content of quartz, possibly resulting in the high quartz abundance under reduced conditions (AQG1/5/6) in this study.

The arid climate and the supply of snowmelt water from the Tianshan mountains are important factors to maintain the weakly alkaline environment in the mud volcano systems [7]. At the same time, the reducing and weakly alkaline medium is beneficial to the formation and precipitation of secondary carbonate minerals such as calcite and siderite in the mud volcano system [1]. However, calcite was only detected in AQG6, whereas there were no clear carbonate mineral characteristic peaks in the other layers. This difference can be explained by the fact that the content of Ca, Mg, and Mn is low, and carbonate mineral precipitation could not occur during the process of hydrocarbons bleaching.

The results of XRF analysis showed distinct differences between the differently colored rock layers. The dissimilarity in the major elements is consistent with the variations in mineral compounds of the corresponding samples, which could reflect the changes in lithology and the environment [43]. From these results, it appears that the rocks were subjected to different degrees of bleaching effect. As the fourth abundant transition metal in the Earth’s crust, iron is widely found in rocks, sediments, and soils in the form of iron oxides and iron-bearing minerals [33], and plays an important role in environmental biogeochemistry. Table 1 indicated that iron is rich in under-oxidized conditions (AQG2/3/4). The decrease in total iron content may imply the dissolution of iron along with the bleaching process. The peak of calcite in AQG6 is due to its highest calcium content (Table 1). Phosphorus content was high under oxidized conditions (AQG3/4). Previous studies have revealed that phosphorus content in rocks is largely related to microbial activities in various natural environments, especially in the presence of iron precipitation [44]. Organic carbon content is known to darken the rock color [8]. LOI values were clearly higher in the gray AQG1 (0.538 wt.%) and the black AQG5 (0.615 wt.%), probably indicating higher contents of organic matter or other types of volatiles absorbed in such layers, which is consistent with previous studies [8]. Together, these results may suggest the contribution of biological processes in the mud volcanic systems [1,7].

### 4.2. Microbial Communities of AQG Terrestrial Rocks

Sediments and minerals can support the growth of a variety of microbial communities, and the composition of microbial communities also feedback, influencing the creation of different environmental conditions [45,46]. As seen in Figure 3C, the abundance of archaea was much smaller than that of bacteria. Overall, the microbial abundance in rocks is relatively less compared to other ecosystems [15,47], possibly due to a lack of nutrients.

Three bacterial phyla, Actinobacteria, and Bacteroidetes were dominant in all rock samples, but their relative abundance differed considerably among the various redox conditions. These phyla are often observed in other extreme environments, and a wide range of relative abundances variations for these phyla across different regions or rock types has also been reported [48]. Actinobacteria, the dominant phylum in our samples, is found widely in aquatic and terrestrial habitats, including extreme habitats such as Antarctic glacier forefields [49] and arid desert soils [50]. Bacteroidetes, most of which are anaerobic bacteria, and can decompose sugars, proteins, and other substrates [51], showed higher relative abundances in the darker layers, such as AQG4 (reddish) and AQG5 (grayish-black). In our rock samples, the relative abundance of Proteobacteria was low, similar to what was observed in other oligotrophic environments [52]. This suggested that fast-growing bacteria would thrive in eutrophic environments, while slower growing bacteria prefer oligotrophic environments [52]. The lower relative abundance of Proteobacteria in our studies may be related to the oligotrophic environment of the high-altitude mud volcano habitats formed in this area [35]. Planctomycetes also showed high relative abundance in certain layers (AQG3). It is a unique phylum of the bacteria that can be found in many different habitats, including oceans, marine sediments, freshwater lakes, wastewater, and terrestrial soils [53]. Acidobacteria occur in other type of rocks, but were relatively rare in our samples, maybe certain bacterial groups had a substrate preference [35].

Many iron-reducing microorganisms could not be classified to the genus levels. As there is no universal primer for iron-reducing bacteria, the known species of iron-reducing bacteria were analyzed according to the published literature [54,55]. A wide phylogenetic diversity of microorganisms are known to reduce iron, and their members include *γ*-, *β*-, *ε*-, *δ*-proteobacteria, and Firmicutes [54]. Iron-reducing bacteria, such as *Pseudomonas* and *Bacillus* were highly enriched in the AQG rock layers. *Bacillus* spp. and *Pseudomonas* spp., which include iron- and manganese-reducing bacteria, have been isolated from a wide variety of environments and are prevalent in soils and sediments [56]. Some *Bacillus* strains are both respirative and fermentative dissimilatory iron-reducing microorganisms. It was found that the main iron-reducing bacteria in the subsurface sediments were also *Bacillus* [57]. In the nature environment, *Escherichia* spp. also have the ability to reduce iron [58,59]. The reduction of insoluble iron oxide is mainly carried out by electron transfer via different membrane-bound and -soluble (i.e., periplasmic) electron carriers, such as *c*-type cytochromes, quinones and multicopper oxidases, may play roles in these systems [60]. The relative abundance distribution of iron-reducing bacteria in sediments may be simultaneously affected by O_2_, sulfate, iron, and other environmental factors, and this complex mechanism requires further study [61]. In general, there was a certain level of variation in community composition among rock layers. These may be due to differences in the chemical composition and microenvironments of the rocks [35]. This differential occurrence of microbial genera depending on the rock’s chemical properties illustrated that redox conditions have a strong control on the distribution of microorganisms [62]. For example, *Brevundimonas* and *Cutibacterium* were dominant in the relative oxidized conditions (AQG2/3/4), while *Halomonas* and *Salinimicrobium* were in high abundance in reduced samples (AQG1/5/6). Phylogeny based on UniFrac is particularly important because they exploit similarities and differences between species, and this additional information makes phylogenetic beta diversity measures more effective than taxon-based approaches in revealing ecological patterns [63]. As can be seen in Figure S4, AQG5 was distinctly different from other layers may be due to its strongly reduced condition. Nevertheless, differences in microbial community composition under different redox conditions are still worth considering.

A so-called “mineralosphere” hypothesizes that the ability of microorganisms to preferentially utilize inorganic nutrients released from the surrounding rocks and that microbial community structure is affected by the surrounding mineral habitats [36]. The role of mineral contents provides more specific insight into the ecological processes shaping anaerobic microbial communities in methane-rich habitats [15]. As shown by Mantel test (Table S4), the total bacterial community was closely linked with the contents of iron, suggesting that in lithic environments, such as our sample rocks, most of the naturally iron compounds are highly insoluble, and therefore act as a limiting nutrient.

### 4.3. The Interaction between Hydrocarbons and Iron-Bearing Minerals Mediated by Microorganisms

Microorganisms are considered to be the most important colonizers of sediments and rocks and are involved in many critical environmental processes, including elemental cycling, mineral transformation, and soil formation [13]. Path analysis fitted the relationship between microbial community, microbial diversity, OTUs richness, iron, and other environmental factors well (*p* > 0.05) (Figure S6A). It can be seen that the microbial community has a significant negative impact on Fe (*p* < 0.05). The diversity of microorganisms (Shannon index) was significantly affected by the microbial community (*p* < 0.01) and positively correlated with the content of Fe (Figure S6A). OTUs richness (Chao index) was affected by the microbial community and negatively correlated with other environmental factors. After considering both direct and indirect influences, the total effect of each factor on iron after standardization is shown in Figure S6B, the effect of microbial diversity (Shannon index) on iron was the largest and positive, which was consistent with the results in Figure 4 and Table 1, where, under oxidized conditions (AQG2/3/4), the microbial diversity was rich, and the iron content was high. In summary, indicators of microorganisms such as microbial community, microbial diversity, and OTUs richness can significantly affect environmental factors (especially Fe) through different pathways (*p* < 0.05). Different microbial communities can reduce the ferric iron to the more soluble ferrous iron, which can then be transported elsewhere [44], thus decreasing the content of total iron in location of observations. The conversion of elements to more mobile forms can lead to a variety of secondary mineral precipitates depending on the different microenvironments, and such formations may contribute to the staining and discoloration of rock and mineral surfaces [13], which in turn may be an important factor in the color stratification of terrestrial sedimentary rocks of the Aiqigou mud volcano.

Many studies have observed microbiological reduction of iron (III) in clay minerals in natural soils [64] and sediments [65,66,67]. Due to the abundant iron content and suitable electrochemical characteristics, the reduction of iron oxides by microorganisms coupled to the oxidation of organic compounds is considered to be a crucial biogeochemical process [68]. Consistent with previous studies, acetate could stimulate the reduction of amorphous ferric oxyhydroxide in enrichments with sediments but not in heat-killed controls [21], suggesting that microbes play an important role in the redox process (Figure 5). With the addition of acetate to anaerobic sediments containing Fe (III) oxide, the reduction of acetate, the increase of CO_2_ produced by acetate, and the increase of Fe (II) occurred at the same time, indicating that iron-reducing bacteria have the potential to completely oxidize organic matter to CO_2_. Enrichment cultivation showed that AQG5 had the highest rate of iron reduction, which may be one of the reasons for its dark coloration. Figure S7 shows the color of the samples in the serum bottle changed after 50 days of incubation. It can be seen that, in the naked eye observation, the color changes of the experimental group are all deeper than those of the control inactivated group, indicating increased content of ferrous iron. In conclusion, there are indeed microorganisms involved in the redox reaction in this area, and Fe, as the main driving factor of the microbial community in this sedimentary rock area, can act as an electron acceptor to affect the anaerobic oxidation process of organic matter in this area. In the Aiqigou mud volcano system, iron-reducing bacteria were found in all layers; Proteobacteria, Firmicutes, and Bacteroidetes are the dominant iron-reducing phyla that appeared in our rock samples. These kinds of iron-reducing bacteria can directly use iron-bearing minerals as terminal electron acceptors to oxidize organics via an electron-shuttling mechanism [68]. Microorganisms involved in this process have the ability to exchange electrons with solid compounds (conductive minerals such as the iron oxide) and organic matter, through a process known as extracellular electron transfer [60,69,70].

In our study, the production of ^12^CH_4_ was lower than the detection limit, which may be due to the addition of ferric iron inhibiting its production in sediments [21]. Because ferric iron is an important electron acceptor prior to CO_2_ utilization in organic anaerobic oxidation process, it can inhibit the production of methane [71]. Additionally, ^13^CH_4_ decreased after 28 days compared with the control group, indicating that microorganisms were involved in the reaction between methane and iron. The electrons obtained from methane oxidation are transferred to iron-bearing minerals by iron-reducing bacteria to complete the reduction of methane and iron oxides [72]. Microorganisms from marine sediments have been reported to use iron (ferrihydrite) for anaerobic oxidation of methane, and anaerobic methanotrophs can complete the above process together with iron-reducing bacteria [73,74]. Subsequently, various forms of iron, such as nanoparticle iron and soluble iron citrate, have been shown to act as electron acceptor coupling for the anaerobic oxidation of methane [75]. In addition, a variety of functional microorganisms, such as methanotrophs and iron-reducing bacteria, have previously been observed to couple oxidation of methane with iron reduction in mud volcanoes [15]. Our results showed the high relative abundance of Thaumarchaeota in all AQG samples, which had been shown to be closely related to the methane cycle [76]. Only two archaea phyla were found in our samples, further studies are needed to reveal the intragenomic variation profile of the archaea domain [77].

**Figure 6:**
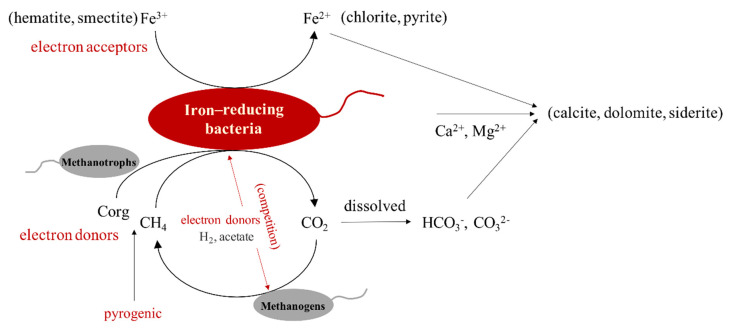
A simplified diagram of organic–inorganic interactions between hydrocarbons and iron-bearing minerals mediated by microorganism in the Aiqigou mud volcano system.

Taken together, the results suggest the presence of a number of microorganisms in the continental sedimentary rocks of the Aiqigou mud volcano could be involved in the reaction between organic compounds and inorganic materials, thus changing the physical and chemical properties of clay minerals and inhibiting methane emissions into the atmosphere, thereby promoting rock color stratification as well as reducing the greenhouse effect. Such redox processes may occur extensively in areas rich in methane and minerals based on previous study [1], as briefly summarized in Figure 6. In summary, analyzing the biogeochemical coupling mechanism between microorganisms and key elements of ecosystem can predict the activity and development trajectory of microbial community in the environment, as well as provide important scientific evidence for the process of historical geological environment change [78].

## 5. Conclusions

In this study, we focused on the bacterial and archaeal communities and their mediated biogeochemical processes in the Aiqigou mud volcano. We combined sequencing information and physicochemical properties to reveal the crucial role of iron as an electron acceptor in shaping microbial community structure in these extreme environments. Simultaneously, we established path analysis to show that microbial communities can also influence environmental factors, especially iron, which may be responsible for the dramatic change in rock color. The impacts of the microorganisms on the interaction between organic matter and iron-bearing minerals were further verified by enrichment experiments, which is the first study on biotic process in different redox environments. This research is valuable for the study of iron biogeochemical processes mediated by iron-reducing bacteria and their partners, and the iron cycle is closely linked to global climate change by affecting CO_2_ and CH_4_ emissions.

## Figures and Tables

**Table 1:** Relative content of the major elements and loss on ignition results of the samples.

Sample ID	SiO_2_ (%)	Al_2_O_3_ (%)	Fe_2_O_3_ (%)	K_2_O (%)	MgO (%)	Na_2_O (%)	TiO_2_ (%)	CaO (%)	MnO (%)	P_2_O_5_ (%)	LOI * (%)
AQG1	62.22	24.75	4.77	2.98	1.83	1.03	1.09	0.426	0.045	0.069	0.538
AQG2	62.39	22.68	6.15	2.66	2.17	1.42	1.03	0.608	0.063	0.076	0.345
AQG3	55.68	22.12	9.76	2.52	2.22	3.18	0.98	0.684	0.191	0.143	0.445
AQG4	57.89	23.62	9.06	3.07	2.07	1.16	0.97	0.816	0.056	0.160	0.517
AQG5	56.61	20.28	5.26	2.48	2.04	6.22	1.02	0.692	0.057	0.074	0.615
AQG6	57.75	27.29	3.90	2.68	2.05	2.52	1.18	0.943	0.073	0.067	0.334

* LOI (loss on ignition) indicates the weight loss during heating at temperature between 80 °C for 4 h and 850 °C for 6 h. XRF can only measure the content of the element, and the results are shown in the form of oxides, which can reflect the relative content of each element.

## Data Availability

The data presented in this study are openly available in the NCBI Sequence Read Archive, the BioProject ID is PRJNA638372.

## Supplementary Materials

The following are available online at https://www.mdpi.com/2076-2607/9/3/560/s1. Table S1: T-test results between each group, Table S2: Physicochemical properties of different sample layers in the Aiqigou mud volcano, Table S3: The multiple comparisons result of HSD test of physicochemical properties of different layers, Table S4: Mantel tests analyses of six sample layers in the Aiqigou mud volcano, Table S5: Individual ANOVA test F and *p* values, Figure S1: Rarefaction curves of six layers, Figure S2: Melting curves of standards and samples, Figure S3: Standard curves of standards and samples, Figure S4: UPGMA clustering tree based on Weighted Unifrac Distance among six layers, Figure S5: Canonical correspondence analysis (CCA) of prokaryotic communities in six layers, Figure S6: A: Path analysis of microbial community, diversity and richness with iron and other environmental factors. B: The total effect of each factor on iron after standardization, Figure S7: The color of the samples in the serum bottle changed after 50 days of incubation.
