# Supplementary material for: Mineralosphere Microbiome Leading to Changed Geochemical Properties of Sedimentary Rocks from Aiqigou Mud Volcano, Northwest China
Source: Microorganisms. 2021 Mar 9;9(3):560. doi: 10.3390/microorganisms9030560 (PMC7998385; doi:10.3390/microorganisms9030560)
Supplement: Supplementary file 1 [file microorganisms-09-00560-s001.pdf]

microorganisms

Supplementary Material

1. Supplementary Tables

Table S1. T-test results between each group

Statistics	Shannon index	Chao index
AQG1 vs. AQG2	*	*
AQG1 vs. AQG3	*	*
AQG1 vs. AQG4	*	*
AQG1 vs. AQG5	ns	*
AQG1 vs. AQG6	*	*
AQG2 vs. AQG3	ns	ns
AQG2 vs. AQG4	ns	ns
AQG2 vs. AQG5	*	ns
AQG2 vs. AQG6	ns	ns
AQG3 vs. AQG4	ns	ns
AQG3 vs. AQG5	*	ns
AQG3 vs. AQG6	ns	ns
AQG4 vs. AQG5	**	ns
AQG4 vs. AQG6	*	ns
AQG5 vs. AQG6	**	ns

(*** $p < 0.001$; ** $p < 0.01$; * $p < 0.05$; ns: $p > 0.05$).

Table S2. Physicochemical properties of different sample layers in the Aiqigou mud volcano.

Sample ID	NH_4^+ (mg kg^{-1})	NO_3^- (mg kg^{-1})	pH	TC (g kg^{-1})	TN (g kg^{-1})	Mn (g kg^{-1})	Fe (g kg^{-1})
AQG1	14.29±0.73	24.90±1.54	7.68±0.02	8.87±0.06	1.08±0.02	0.26±0.01	25.09±0.44
AQG2	7.90±0.15	22.05±0.35	7.71±0.05	2.41±0.02	0.43±0.02	0.42±0.03	34.22±1.32
AQG3	2.77±0.25	144.07±4.67	8.42±0.06	1.13±0.08	0.58±0.01	1.34±0.01	50.66±0.10
AQG4	15.66±0.25	62.91±3.87	7.99±0.04	3.03±0.04	0.89±0.01	0.37±0.00	48.52±0.27
AQG5	9.35±0.03	231.94±2.09	7.51±0.07	8.49±0.05	1.06±0.01	0.37±0.01	30.51±0.34
AQG6	0.97±0.04	43.36±0.33	8.83±0.05	4.82±0.02	0.33±0.00	0.46±0.02	17.88±0.70

The numbers in the table represent means plus/minus standard deviations.

TC: total carbon, TN: total nitrogen.

Table S3. The multiple comparisons result of HSD test of physicochemical properties of different layers

Statistics	NH_4^+	NO_3^-	pH	TC	TN	Mn	Fe
AQG1 VS. AQG2	***	ns	ns	***	***	***	***
AQG1 VS. AQG3	***	***	***	***	***	***	***
AQG1 VS. AQG4	***	***	***	***	***	***	***
AQG1 VS. AQG5	***	***	**	***	ns	***	***
AQG1 VS. AQG6	***	***	***	***	***	***	***
AQG2 VS. AQG3	***	***	***	***	***	***	***
AQG2 VS. AQG4	***	***	***	***	***	**	***
AQG2 VS. AQG5	***	***	***	***	***	**	***
AQG2 VS. AQG6	***	***	***	***	***	**	***
AQG3 VS. AQG4	***	***	***	***	***	***	**
AQG3 VS. AQG5	***	***	***	***	***	***	***
AQG3 VS. AQG6	***	***	***	***	***	***	***
AQG4 VS. AQG5	***	***	***	***	***	ns	***
AQG4 VS. AQG6	***	***	***	***	***	***	***
AQG5 VS. AQG6	***	***	***	***	***	***	***

The values show the significant levels: * $p < 0.05$; ** $p < 0.01$; *** $p < 0.001$; ns: $p > 0.05$.

TC: total carbon, TN: total nitrogen.

Table S4. Mantel tests analyses of six sample layers in the Aiqigou mud volcano.

	Simple Mantel		Partial Mantel	
Envs	*r*	*p*	*r*	*p*
NH_4^+	0.251	0.005	-0.057	0.742
**NO_3^-**	0.420	**0.001**	0.401	**0.001**
pH	0.248	0.008	-0.093	0.854
TC	0.326	0.003	0.059	0.211
TN	0.195	0.018	-0.200	0.997
Mn	0.284	0.01	0.160	0.055
**Fe**	0.477	**0.001**	0.350	**0.001**

The bold represents $p < 0.05$ under Partial Mantel analysis.

TC: total carbon, TN: total nitrogen.

Table S5. Individual ANOVA test F and *p* values.

	**NO_3^-**	pH	TN	**Mn**	**Fe**
F	2.318	1.527	1.433	2.157	2.523
p	**0.001**	0.070	0.093	**0.015**	**0.001**

The bold represents $p < 0.05$.

TN: total nitrogen.

2. Supplementary Figures

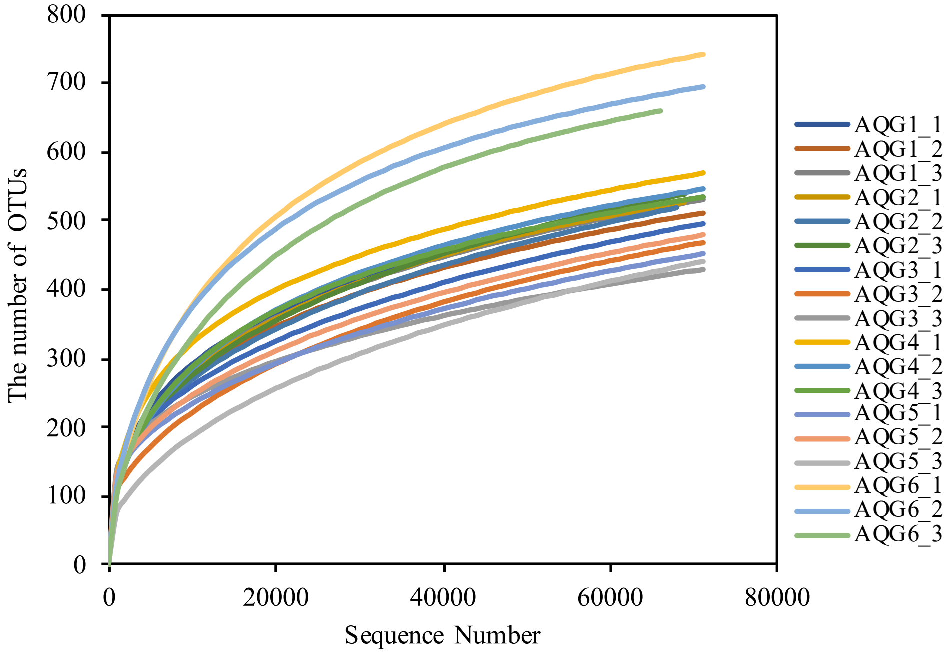

Figure S1. Rarefaction curves of six layers. The rarefaction curves were demonstrated sampling efficiency of the microbial 16S rRNA gene sequences.

A

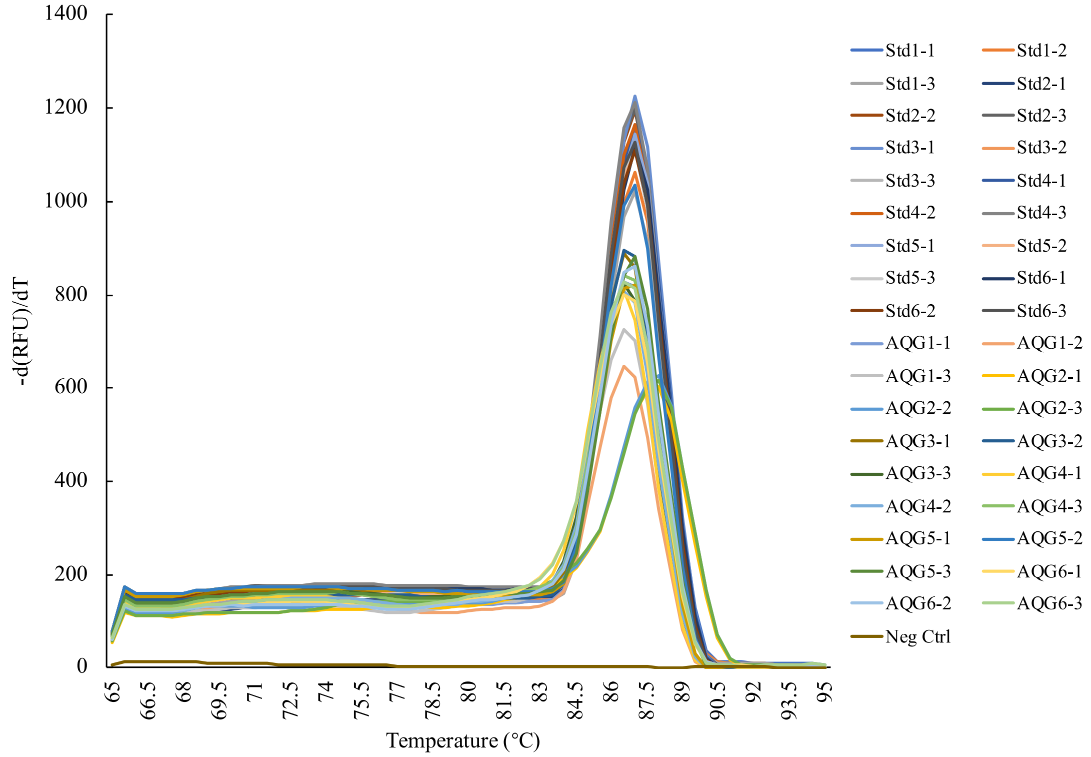

B

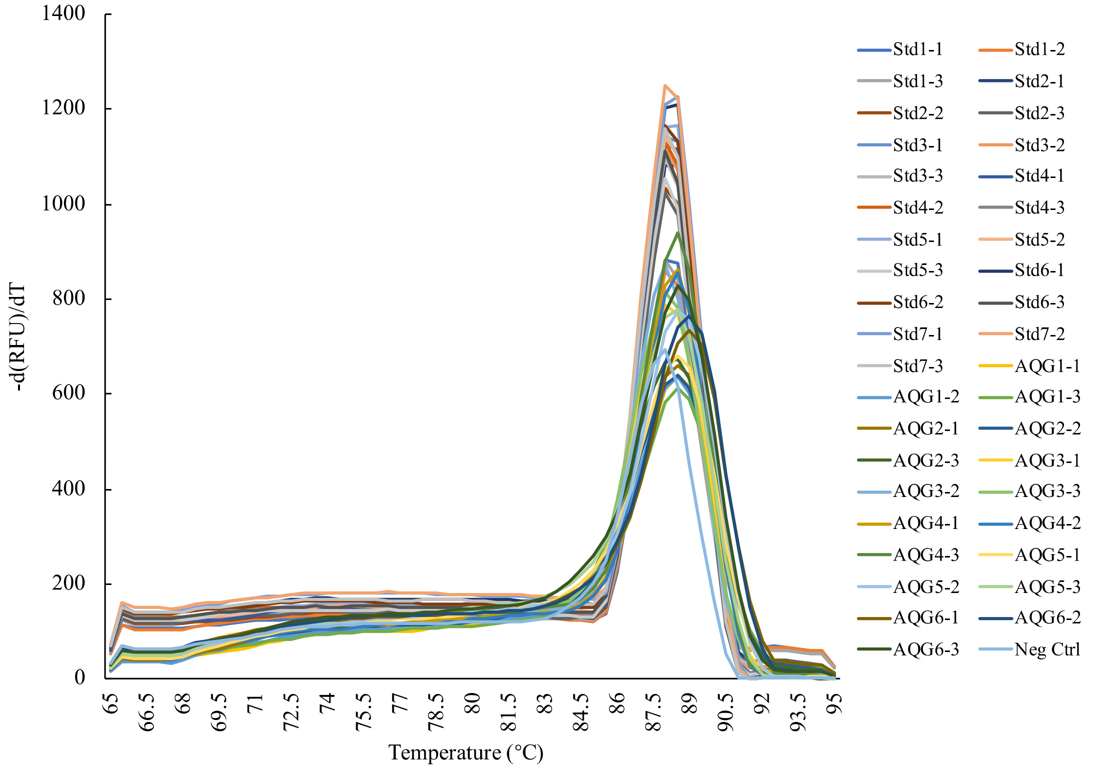

Figure S2. Melting curves of standards and samples. A: archaea (E = 95.7%), B: bacteria (E = 109.1%).

Std: standard samples, Neg Ctrl: negative control.

A

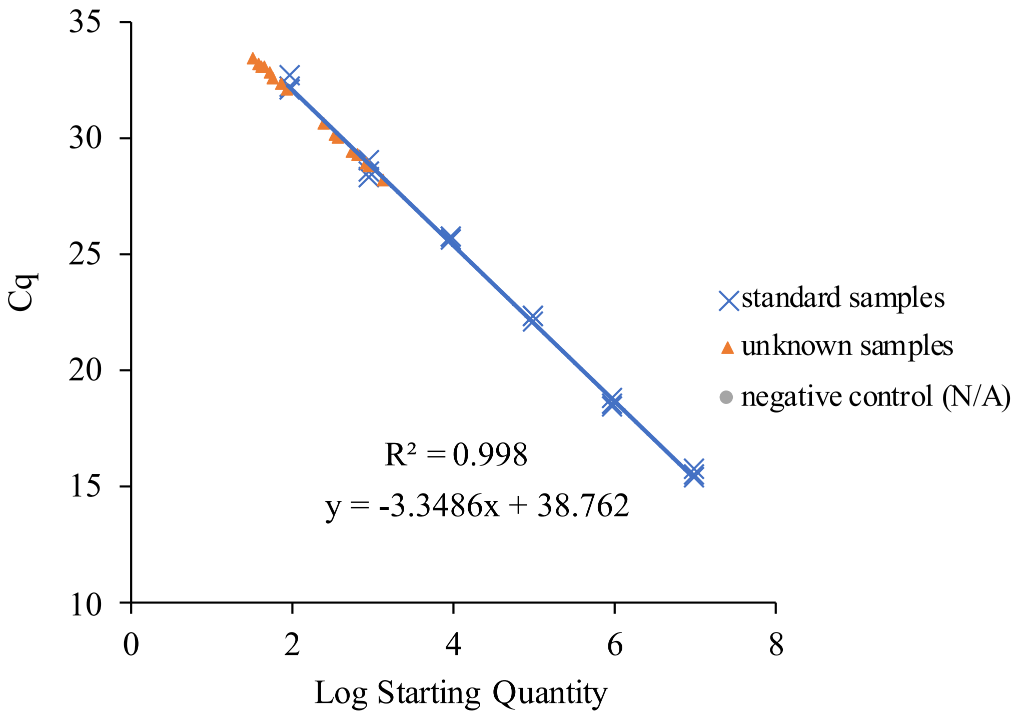

B

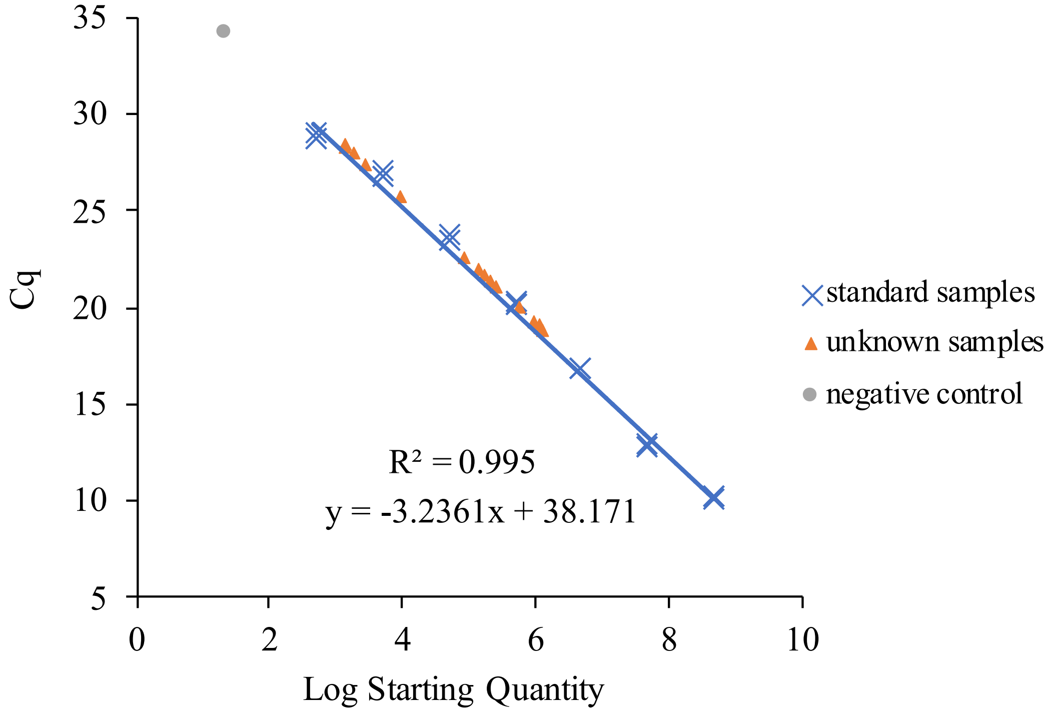

Figure S3. Standard curves of standards and samples. The line in the figure were drawn based on the results of standard samples. R^2 and the equations are shown in figure. A: archaea, B: bacteria.

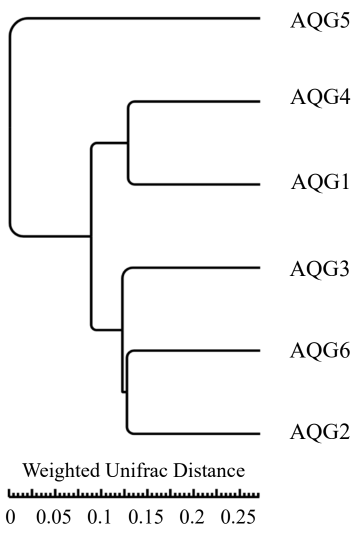

Figure S4: UPGMA clustering tree based on Weighted Unifrac Distance among six layers. The value indicates the means of 3 replicates.

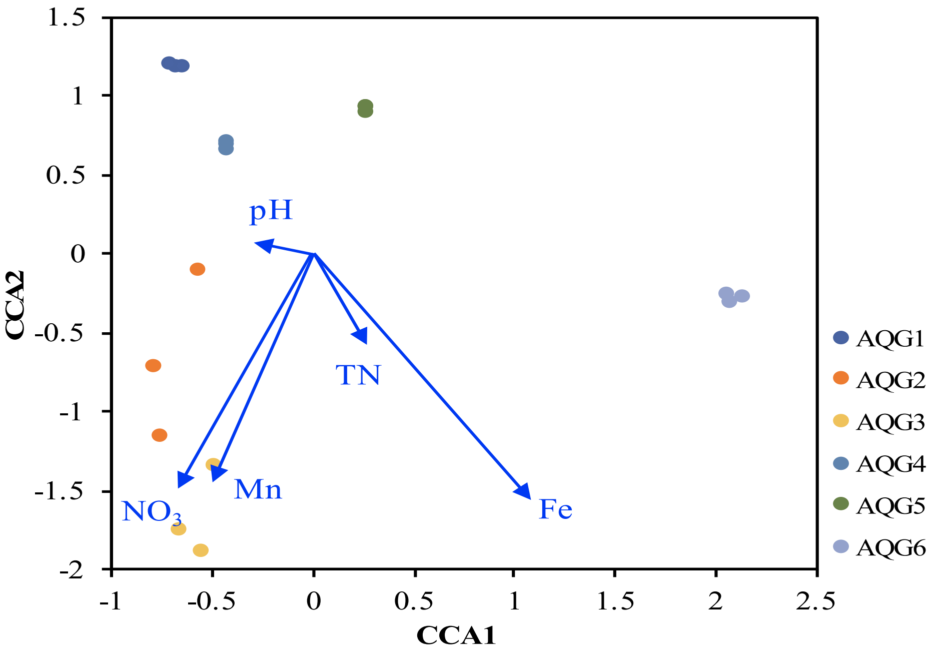

Figure S5. Canonical correspondence analysis (CCA) of prokaryotic communities in six layers. Different colored circles represent different layers.

A B

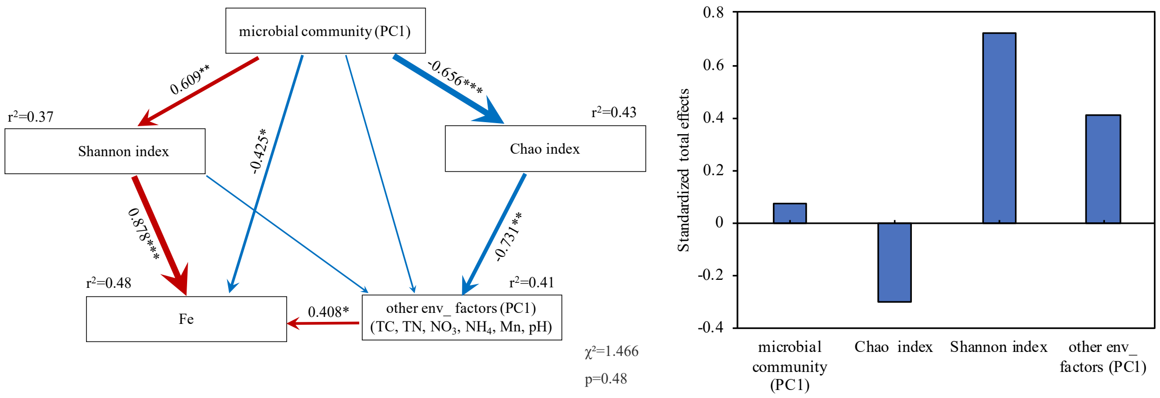

Figure S6. A: Path analysis of microbial community, diversity and richness with iron and other environmental factors. The number on the arrow indicates the standardized impact factor, with the asterisk indicating significance (*** $p < 0.001$, ** $p < 0.01$, * $p < 0.05$). The red arrow indicates the positive effect, the blue arrow indicates the negative effect, and r^2 represents the degree of interpretation of the factor. B: The total effect of each factor on iron after standardization.

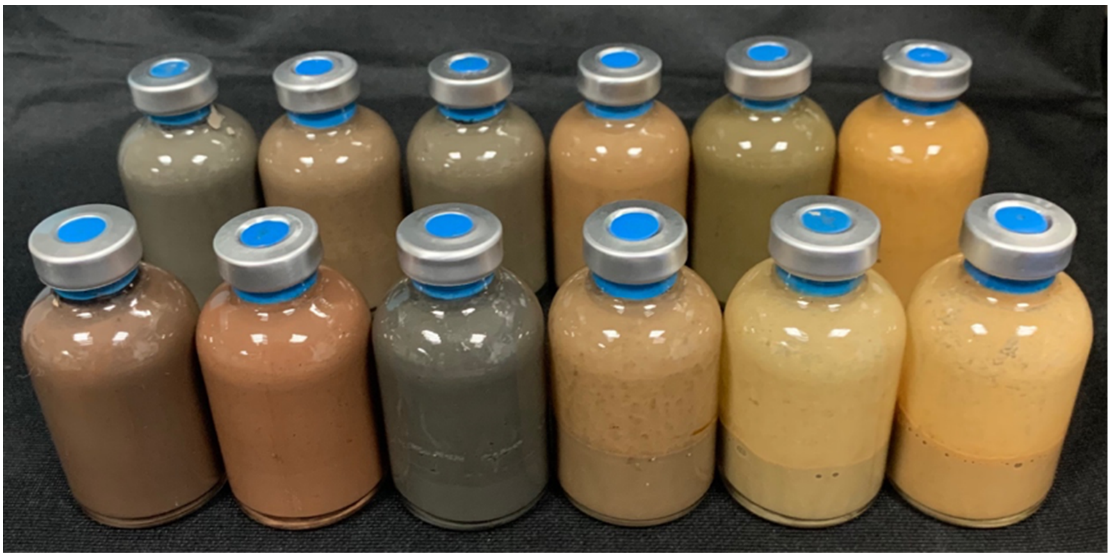

Figure S7. The color of the samples in the serum bottle changed after 50 days of incubation. From left to right, from top to bottom: AQG1 experimental group and inactivated group, AQG2 experimental group and inactivated group, AQG3 experimental group and inactivated group, AQG4 experimental group and inactivated group, AQG5 experimental group and inactivated group, AQG6 experimental group and inactivated group.
